# Degenerative Suspensory Ligament Desmitis (DSLD) in Peruvian Paso Horses Is Characterized by Altered Expression of TGFβ Signaling Components in Adipose-Derived Stromal Fibroblasts

**DOI:** 10.1371/journal.pone.0167069

**Published:** 2016-11-30

**Authors:** Wei Luo, John Sandy, Katie Trella, Daniel Gorski, Shuguang Gao, Jun Li, Sabrina Brounts, Jorge Galante, Anna Plaas

**Affiliations:** 1 Department of Internal Medicine, Rush University Medical Center, Chicago, IL, United States of America; 2 Department of Orthopedic Surgery, Rush University Medical Center, Chicago, IL, United States of America; 3 Department of Bioengineering, University of Illinois at Chicago, Chicago, IL, United States of America; 4 Department of Biochemistry, Rush University Medical Center, Chicago, IL, United States of America; 5 Department of Surgical Sciences, University of Wisconsin-Madison, School of Veterinary Medicine, Madison, Wisconsin, United States of America; University of Alabama at Birmingham, UNITED STATES

## Abstract

Equine degenerative suspensory ligament desmitis (DSLD) in Peruvian Paso horses typically presents at 7–15 years and is characterized by lameness, focal disorganization of collagen fibrils, and chondroid deposition in the body of the ligament. With the aim of developing a test for disease risk (that can be used to screen horses before breeding) we have quantified the expression of 76 TGFβ-signaling target genes in adipose-derived stromal fibroblasts (ADSCs) from six DSLD-affected and five unaffected Paso horses. Remarkably, 35 of the genes showed lower expression (p<0.05) in cells from DSLD-affected animals and this differential was largely eliminated by addition of exogenous TGFβ1. Moreover, TGFβ1-mediated effects on expression were prevented by the TGFβR1/2 inhibitor LY2109761, showing that the signaling was via a TGFβR1/2 complex. The genes affected by the pathology indicate that it is associated with a generalized metabolic disturbance, since some of those most markedly altered in DSLD cells (*ATF3*, *MAPK14*, *ACVRL1 (*ALK1), *SMAD6*, *FOS*, *CREBBP*, *NFKBIA*, *and TGFBR2*) represent master-regulators in a wide range of cellular metabolic responses.

## Introduction

Degenerative suspensory ligament desmitis (DSLD) is prevalent in specific horse breeds, such as the Peruvian Paso, Paso Fino, American Saddlebred, Quarter Horse, and Akhal-Teke [1, 2] and is rare in pony and draft breeds amongst others. In specific Peruvian Paso families, prevalence is up to 40% [2]. The onset of DSLD is subtle and manifests itself with progressive lameness in multiple limbs in the absence of trauma. The age of the horse at diagnosis is usually approx. 7–15 years [3]. Very seldom is it seen in horses older than 15 years of age. Fetlock hyperextension and suspensory ligament (SL) swelling develop over time as well as scar tissue surrounding the suspensory ligament. Clinically the horses present with only the suspensory ligament affected, and no detectable gross changes in flexor tendons or even extensor tendons have been reported. It has been suggested that DSLD may be a systemic disorder involving all tissues and organs [1], but with a more significant connective tissue component. This may emerge more distinctly, due to the more extensive mechanical stressors on the affected SLs.

The diagnosis of DSLD is based upon signalment, history, clinical signs, and imaging [4]. Affected SLs show diffuse loss of echogenicity, an irregular fiber pattern, and diffuse enlargement ultrasonographically [3, 4]. Histological and biochemical studies of DSLD [1, 5] have revealed many similarities to human [6] and murine [7] tendinopathies including the disorganization of collagen fibrils [1, 8, 9] and the presence of hyperplastic aggrecan-rich chondroid deposits [10].

The pathogenesis of DSLD is not well understood. Generalized accumulation of proteoglycans in connective tissue has been considered important. Accumulation (~15-fold above normal) in SLs of distinct aggrecan fragments and hyaluronan suggested defective ADAMTS-aggrecanase (A-disintegrin-and-metalloproteinase-with-thrombospondin-like-motifs)-5 function [5]. Notably, murine stromal cells from *Adamts5 -/-* mice [11] have shown that aggrecan accumulation occurs in the absence of ADAMTS5, not because of decreased aggrecan degradation, but apparently because a specific fragment of the protein down-regulates (via a non-proteolytic mechanism) cellular glucose uptake via GLUT4. The excess supply of glucose (and ATP synthesis) in the *Adamts5 -/-* mouse results in high rates of chondroitin sulfate/aggrecan synthesis and tissue deposition which, in turn, has been shown to have adverse effects on tendon mechanical properties [12]. Although changes to the *ADAMTS5* gene or protein might contribute to the DSLD genotype, it seems likely that DSLD is a complex trait in which habitual athleticism and ageing also influence disease risk [2]. Improved understanding of the disease process of DSLD is therefore needed. Affected horses have typically been used for breeding before clinical diagnosis of DSLD. There is, therefore, a need to develop a test for DSLD risk that can be used to screen horses before breeding.

While SNP variants of matrix proteins in DSLD-Pasos have not been reported, recent analysis of 270 racehorses with superficial digital flexor tendinopathy (SDFT) has suggested (odds ratio, 2.77) an association with a G >A substitution in the *COL5A1* gene [13], which, along with variants in other genes, such *COL27A1*, *CASP8*, *MMP3*, *FBN2*, *TIMP2*, has been implicated in matrix changes in various human tendinopathies [14]. A genetic predisposition to defective matrix organization in human tendinopathy is also supported by a recent bioinformatics analysis [15], which identified four strong candidate risk genes (*COL11A2*, *ELN*, *ITGB3*, *LOX*), each of which functions in the controlled turnover of fibrous tissue matrix. In addition, in our own studies, DNA methylome analysis of experimental murine tendinopathy [16] has implicated dysregulation of *Leprel2* (Prolyl3-hydroxylase-3), an enzyme which hydroxylates proline in the 3-position and which appears to control collagen fibril diameter specifically in tendon [17].

With a view to molecular diagnosis and mechanistic understanding of DSLD we have now taken two new approaches. In the first, we analyzed ligament tissues from DSLD-Pasos, NA-Pasos (Non Affected Pasos) and Non-Pasos for expression of matrix genes (*ACAN*, *FN1*, *COL1A1/A2*, *COL3A1*, *COL5A1/A2/A3*, *and TGFB1*). In the second we have quantified the expression of 80 TGFb-signaling target genes in adipose-derived stromal fibroblasts (ADSCs) from DSLD and NA-Paso horses. The rationale for this approach was based on our finding [11] that culture-maintained newborn dermal fibroblasts [18] and ADSCs from mature wild-type and knockout mice [11] continue to exhibit altered gene expression profiles and metabolic responses that reflect the pathological matrix remodeling seen during dermal wound-healing and Achilles tendon repair in vivo. Analysis of transcript abundance of TGFβ1 signaling target genes was chosen because firstly, severe murine tendinopathy can be induced by intra-tendinous injection of TGFβ1 [7], secondly, human tendinopathy has been linked to changes in TGFβ1 expression and activation [19] and thirdly, the appearance of DSLD tendons (macroscopically and microscopically) indicates deposition of a scar-like tissue (akin to a TGFβ1-induced fibrosis) within the ligament body itself and in the surrounding tissues.

## Methods

### Animals

Horses were donated and owners of all horses signed a donation and euthanasia consent form, and were used for teaching purposes under the Animal Care and Use Committee of the University of Wisconsin approved protocol V00348. After euthanasia with sodium pentobarbital tissue procurement was carried out at the School of Veterinary Sciences, U Wisconsin. Tissue procurement procedures on euthanized horses were exempt from an RARC permit.

A detailed description of the breed, age, and sex of each horse are provided in S1 Table. Eleven horses were Peruvian Pasos and six were other breeds. Female horses were not pregnant at the time of collection. Horses for SL tissue donation were euthanized for reasons unrelated to any tendon or ligament disease (non-Paso group, n = 5 and NA- Paso group, n = 2) or for being affected with DSLD (diagnosed, n = 6) (S1 Table).

Each horse received a physical examination with palpation of soft tissue structures and a gait evaluation at a walk and trot in hand on a soft and hard surface. In addition, ultrasonography of the palmar/ plantar aspect of each limb was performed using a linear transducer at a frequency of 10 MHz to evaluate the tendons and suspensory ligaments. Transverse and longitudinal still images of the SL at 5cm, 10cm, and 15cm distal to the accessory carpal bone in forelimb or 10cm, 15cm and 20cm distal to point of the hock in hind limb as well as the lateral and medial branches of SL were obtained to confirm normal or abnormal tendon and suspensory ligament structures. Based on these findings the horses were classified into the Non-Paso, NA-Paso and DSLD-Paso groups.

### Harvest of Suspensory Ligaments and Processing for QPCR analyses

Harvest of suspensory ligament tissue was performed within 15–30 minutes of euthanasia. The palmar/plantar aspect of each limb was clipped and aseptically prepped for tissue collection. A sharp 15–20 cm longitudinal incision was made over the palmar/plantar aspect of the limb and the annular ligament, the superficial and deep digital flexor tendons were dissected off to expose the SL and its branches. A 9 cm long portion of the SL body (extending proximal from the branch point) from both limbs was dissected away from surrounding tissue. This region of the SL is always thicker in diameter and has more scar tissue around it than for healthy SLs. SL samples were immediately placed into RNALater (ThermoFisher) and stored frozen. The typical swollen appearance of DSLD ligament, relative to NA-Pasos (Normal) is shown in “**S1 Fig”** (panel A). Ultrasound images (panel B) further illustrate the swelling and disorganization of the linear collagen bundles.

For analysis, each SL was thawed on ice divided in half lengthwise and each half was divided transversely into one-third portions, termed Part A (most proximal three cm), Part B (most distal three cm and therefore closest to the branch point) and Part C (central three cm). Each SL, whether from the right hind leg (termed RH) or left (termed LH) gave rise to six portions (of about 400mg wet wt.), however not all portions from each SL were analyzed. These individual pieces were quick frozen in liquid N2, then fragmented by hammer impact at -196°C in a Bessman Tissue Pulverizer, recovered, and extracted in 1 mL of Trizol (ThermoFisher) by vortexing for 60 seconds. RNA was prepared using an RNeasy MiniKit (Qiagen), yielding A260/A280 purity of >1.98. cDNA synthesis was performed with 1000 ng of RNA using the SuperScript III First Strand Kit (Qiagen). For individual QPCR assay, Taqman primers were obtained from Qiagen, for the following genes: *COL1A1*, EC03469673_m1; *COL1A2*, EC03469522_m1; *COL3A1*, Ec03469743_m1; *COL5A1*, AJBJX5H; *COL5A2*, SS03389488_G1; *COL5A3*, AJCSWBR; *ACAN*, Ec03469667_m1; *FN1*, Ec03470761_m1; *TGF*β*1*, Ec03468034_m1; *TGF*β*2*, Oc03398420_m1; *TGF*β*3*, Rn00682163_m1).

### Isolation of adipose-derived stromal fibroblasts (ADSCs) and processing for cultures

Harvest of subcutaneous adipose tissue was performed within 15–30 minutes of euthanasia. For non-euthanized horses, an outpatient surgical procedure under local infiltration anesthesia and sedation in the hospital was performed. A 15x15 cm square on the hind end was clipped on either the left or right side of the tail head and aseptically prepped for tissue collection. A sharp longitudinal 5–10 cm incision was made through the skin to the level of the subcutaneous adipose tissue. The adipose tissue was sharply dissected off the surrounding tissue so that a strip of approx. 5 cm long and 1–2 cm thick could be collected. The site was routinely closed if the horse stayed alive. Immediately after collection, tissues were place in cold CO_2_ independent media (ThermoFisher) supplemented with 50 μg/mL Plasmocin (InVitrogen), 5000 U/mL Penicillin, 500 ug/mL Streptomycin (ThermoFisher) and 5 mM glucosamine. HCl (Sigma) and transported on ice to the laboratory. ADSCs were isolated as described [11] within 18 hours of tissue collection. Briefly, tissue was washed in PBS containing antibiotics to remove adherent lipid, then diced into 2 mm cubes and digested with collagenase (Type II, Worthington, 4 mg/mL in CO2 independent medium, 5 mL per g of tissue) for 2.5h at 37°C with agitation. Stromal cells were separated from adipocytes by centrifugation, the pellet treated with Red Blood Cell Lysis Buffer (Sigma Aldridge), washed 3 times with 50 mL of PBS and plated at 1.5–2.0 x10^6 cells per T25 flask in medium A (DMEM with 10% fetal bovine serum (Atlanta Biologics), 5 mM glucose, 2 mM glutamine and antibiotics). At confluence, cells were passaged using trypsin detachment and replated at 1 x 10^6^ cells per T25 flask in medium A and at the 2nd passage cells were trypsinized and plated at 0.5 x 10^5^ cells per well in 12-well plates in medium A. At ~ 90% confluence **(“S2 Fig”)** cultures were changed into medium B (AMEM (Gibco Lifetechnologies) containing 2% FBS (Atlanta Biologics), 2 mM glutamine, 5 mM glucose and Pen/Strep. Advanced MEM is DMEM supplemented with ethanolamine, glutathione, ascorbic acid, insulin, transferrin, AlbuMAX I lipid-rich bovine serum albumin, sodium selenite, ammonium metavanadate, cupric sulfate, and manganous chloride.

Cultures were then treated for an additional 24 hours in medium B +/- 10 ng/mL active TGFβ1. Since TGFβ1 stimulation of cells was carried out under reduced serum conditions, maintenance of cell viability and metabolic responsiveness necessitated the use of this specially formulated AMEM medium **[11]**. It should also be noted that fetal bovine serum has been reported to contain TGFβ protein ranging from 10–20 ng/mL **[20, 21]**, however it is unknown what proportion of this is in a bioactive form. During the expansion of the cells (in 10% FCS), cultures were exposed to TGFβ1 protein is between 1–2 ng/mL, whereas in the treatment period with 10 ng active TGFβ1 protein (in 2% serum), the contribution of endogenous protein would be 0.2–0.4 ng/mL.

Additional cultures were treated with or without TGFβ1 and 30 nM TGFβ Receptor I/II inhibitor, (LY2109761; Selleck). The typical appearance of cultures from DSLD-Pasos and NA-Pasos in untreated, TGFβ1-treated and TGFβ1+ LY2109761 cultures are shown in “**S2 Fig”.**

The experiment on the effect of LY2109761 on basal expression of target genes was done with cells that had been preserved in liquid N2 (in FBS: DMSO, 95:5, v: v) after the first passage. Thawed cells were plated at 1x106 cells per T25 flask in medium A, grown to confluency and subcultured into 12 well plates in medium A and maintained as described for primary cultures. For gene expression assays, cells were maintained with or without inhibitor in medium B for 24 hours and harvested for RNA isolation as described below.

We did not examine the equine ADSC cultures for cell surface ‘marker’ expression, as we previously described for the equivalent cell type from murine adipose **[11]**. However, passaged monolayer growth conditions and the morphological appearance of the cells **(“S2 Fig”**) were consistent with a predominantly fibroblastic phenotype. Furthermore, a study with 2 separate preparations of equine ADSCs for induction of chondrogenic expression in pellet culture (as described in **[22]**, failed to produce activation of chondrogenic genes or accumulation of an ECM enriched in aggrecan and collagen II (data not shown).

For QPCR analysis, 1 mL of Trizol was added to each well, cell layers dispersed and lysed by pipetting, and extracts stored at -20C until further processing. RNA was purified using the RNeasy MiniKit (Qiagen) and for SYB green array QPCR, cDNA was synthesized with 500 ng of RNA using the RT2 First Strand Kit (Qiagen). RT2 Profiler Custom-made array plates for equine TGFβ1 signaling targets and chromatin modification enzymes were from Qiagen and the gene lists are in S2 and S3 Tables respectively.

### Data Calculation and Statistical Analyses

Changes in ΔCt (Ct for transcript of interest minus Ct for Gapdh, (values from 19.04–20.57) were used to calculate the mRNA transcript abundance (2^-∆Ct x 1000). Any gene exhibiting a Ct >34 was considered not detected (ND). Each QPCR assay of individual genes was conducted in triplicate and shown to have a coefficient of variation of less than 7%. While each RNA sample was analyzed on a single TGFβ signaling array plate, data from single samples analyzed on three separate plates have shown a coefficient of variation of less than 5%. To determine the significance of differences in average ΔCt values (triplicate analysis) for individual genes in ligament tissue, 1-way ANOVA with Tukey's post-hoc tests for individual comparisons was conducted using GraphPad Prism 5.

In cell experiments, the average ΔCt value (n = 2–3 cultures) was used to calculate transcript abundance (2^-∆Ct x 1000) in each condition (DSLD-Paso or NA-Paso +/- TGFβ1 +/- LY2109761). These values were used to calculate “fold change” or “fold-difference” as plus (high abundance/low abundance) for an increase or minus (high abundance/low abundance) for a decrease. Student’s t-tests using GraphPad Prism 5 was used on ΔCt values to determine the significance (p<0.05) in expression of genes in TGFβ1 treated groups compared to non-treated groups for both DSLD and NA-Paso cells. Statistical analysis compared data from six DSLD-Pasos (duplicates, n = 12 cultures) and five NA- Pasos (duplicates, n = 10 cultures)

## Results

### Expression of matrix genes in multiple portions of suspensory ligaments from NA-Pasos, DSLD-Pasos and Non-Pasos

**Fig 1:**
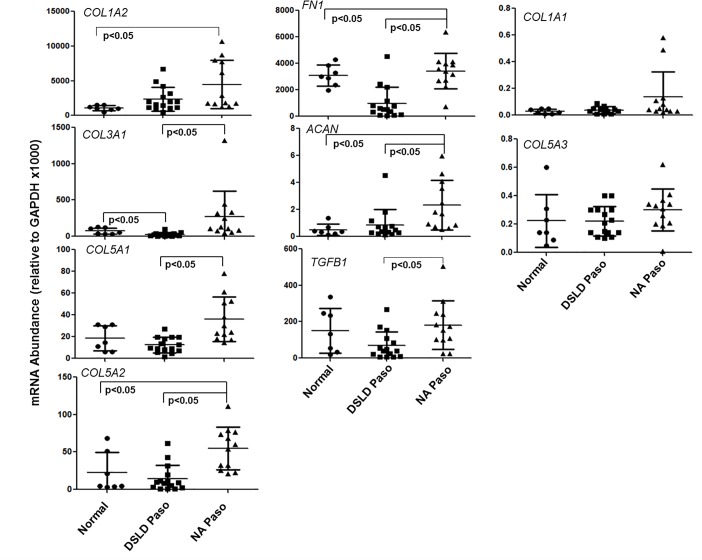
Transcript abundance of ECM remodeling genes in suspensory ligaments. Normals n = 8, DSLD-Pasos n = 15 and NA-Pasos n = 12. Statistical analyses were performed as describe in the Methods Section. Details of horses used for the tissue collection are outlined in S1 Table.

Details of the horses from which SLs were collected for this analyses are provided in **S1 Table**, and expression of matrix genes in multiple portions of ligaments from four DSLD-Pasos, two NA-Pasos and four Non-Pasos, is shown in “**Fig 1”**.

All samples showed essentially the same hierarchy of transcript abundance (COL1A2 ~ FN1 > TGFβ1 > COL3A1 > COL5A1 ~ COL5A2 > ACAN > COL5A3 > COL1A1), consistent with the tissue purity of all ligament portions analyzed. A comparison of the mean expression values for each gene in the three groups (data combined from all locations and all horses in the group Non-Pasos (N = 7), DSLDs (N = 15) and NA-Pasos (N = 12) showed group-specific differences (p<0.05) for all genes except COL1A1 and COL5A3 (“**Fig 1A”**). Thus, the data indicated that the expression of FN1, TGFβ1, COL3A1, COL5A1, COL5A2, and ACAN was similar in Non-Pasos and DSLDs but higher (p<0.05) in NA-Pasos. However, the statistically higher values for the NA-Paso group were largely due to the data for one horse (# 5, “**S1 Table”**), except for TGFβ1 where the higher values were in horse #4, so that when the group comparisons were made between animals (Non-Pasos (N = 4), DSLDs (N = 14) and NA-Pasos (N = 2), the differences did not reach statistical significance. Nonetheless the data suggested that the expression of matrix genes might be used to distinguish between horses in these groups. However, since we had limited access to fresh ligament samples of this kind for the current study, and were unable to expand our SL tissue-based analyses, we decided to use readily available ADSCs, where sampling does not require euthanasia and age-related longitudinal studies are possible.

Studies on a TGFβ1-induced murine tendinopathy model [16] showed that initiation and progression of the pathology was accompanied by marked changes in the expression of chromatin-modification enzymes. We therefore assayed the RNA samples from “**Fig 1”** on custom arrays for expression of epigenetic modifiers (see gene list **“S2 Table”**). These assays (“**S5 Table”)** showed no statistical differences between NA-Pasos and DSLD-Pasos, however there appeared to be a trend toward higher expression in DSLD-Pasos, in contrast to the lower (p<0.05) expression of matrix genes in DSLD-Pasos.

### ADSCs from DSLD-Pasos exhibit markedly lower expression of TGFβ-signaling target genes relative to cells from NA-Pasos

**Fig 2:**
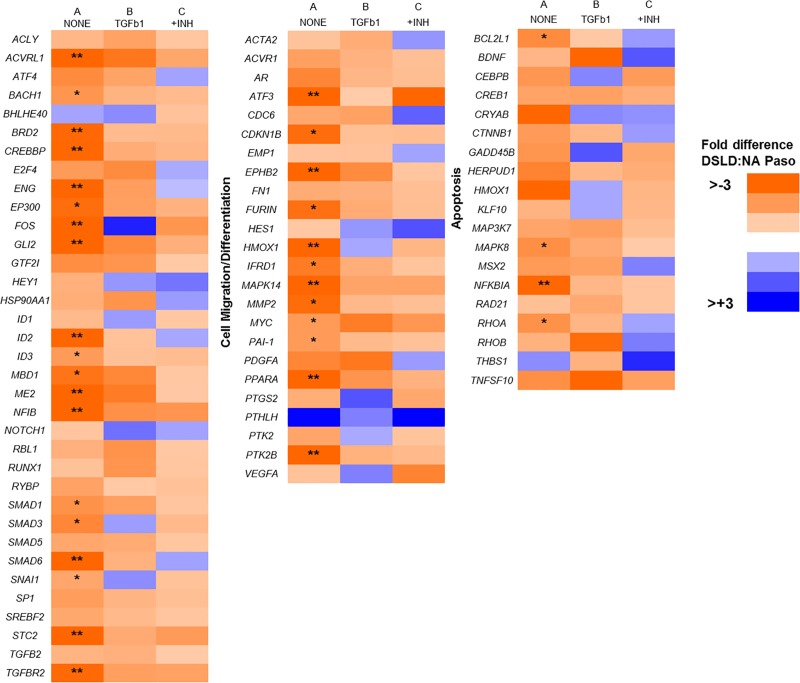
Heat map of fold-difference in transcript abundance of TGFβ signaling target genes in primary ADSC cultures from DSLD-Pasos and NA-Pasos. Statistical evaluation was performed as outlined in the Methods. Statistically significant decreases (p<0.05) in expression in DSLD Pasos is indicated: ** > 3 fold decreased; *1.5–3 fold decreased.

Assay of ADSC cultures from NA-Pasos (n = 5) and DSLD-Pasos (n = 6) showed **that** the transcript abundance of 76 TGFβ1-target genes was lower in DSLD-Pasos than in NA-Pasos (see “**Fig 2”** heat map for fold-differences) and this difference was statistically significant (p<0.05) for the 35 genes listed in “**Table 1”** (see Data Calculation and Statistical Analyses in Methods for calculation of positive and negative fold-differences).

**Table 1:** TGFβ-signaling target genes with low expression in ADSCs from DSLD-Pasos.

	mRNA Abundance^1^		Fold Difference
*Genes*	DSLD Paso	NA Paso	DSLD Paso:NA Paso
*PTK2B*^*2*^	0.62	3.40	-5.47
*ATF3*	0.67	3.32	-4.93
*MAPK14*	8.95	42.98	-4.80
*ME2*	5.46	24.50	-4.49
*ACVRL1*	0.29	1.19	-4.15
*NFIB*	18.82	77.71	-4.13
*EPHB2*	8.93	34.93	-3.91
*HMOX1*	29.83	102.5	-3.44
*SMAD6*	4.90	16.01	-3.27
*FOS*	4.37	13.90	-3.18
*GLI2*	0.46	1.47	-3.17
*STC2*	5.65	17.36	-3.07
*ID2*	46.71	142.4	-3.05
*PPARA*	2.81	8.52	-3.03
*ENG*	39.98	120.1	-3.01
*CREBBP*	12.45	37.07	-2.98
*NFKBIA*	10.32	30.59	-2.97
*BRD2*	31.83	93.94	-2.95
*TGFBR2*	36.85	107.6	-2.92
*MMP2*	313.7	903.0	-2.88
*CDKN1B*	14.11	40.49	-2.87
*EP300*	9.05	25.67	-2.84
*FURIN*	1.05	2.92	-2.79
*MBD1*	3.80	10.34	-2.72
*IFRD1*	7.18	18.39	-2.56
*SMAD3*	21.93	51.49	-2.35
*BCL2L1*	5.78	13.05	-2.26
*MAPK8*	4.42	9.65	-2.18
*SMAD1*	8.72	18.60	-2.13
*RHOA*	137.6	293.5	-2.13
*BACH1*	9.81	20.10	-2.05
*MYC*	33.04	65.00	-1.97
*PAI-1*	351.9	686.8	-1.95
*ID3*	213.64	415.4	-1.94
*SNAI1*	50.64	87.37	-1.73

^1^Mean abundance data for NA-Pasos (n = 5) and DSLD-Pasos (n = 6) is provided (see S1 Table for animal details). Calculation of statistical significance and abundance values are given in the methods Section.

^2^Genes are listed in order of fold-difference between DSLD-Pasos and NA-Pasos.

This hierarchy provided some insight on the function of the genes most inherently affected by the presence of the DSLD pathology. Thus, of the 19 most-affected genes (> minus 2.9-fold difference) their functions are classified on the array as “signal transduction” (n = 13), “cell migration and differentiation” (n = 5) and apoptosis (n = 1). Of the 13 signal transduction genes, five (*ACVRL1*(ALK1), *TGFBR2*, *ENG* (TGFBR3), *SMAD1* and *SMAD3*) are components of the TGFβ receptor and SMAD signaling networks suggesting that the apparent “deficiency” in DSLD cells might be due to the altered expression of one or more of these network components.

### TGFβ1 treatment inhibits expression of TGFβ1-target genes more profoundly in ADSCs from NA-Pasos than from DSLD-Pasos

We next examined the effect of TGFβ1 (10ng/ml) on expression of the 35 genes (**in Table 1**) in ADSC cultures. The data (**Table 2**) shows that TGFβ1 had a general inhibitory (rather than the expected stimulatory) effect on expression in both cultures (as shown by the negative values in the “fold-effect of TGFβ1” columns). Notable exceptions to this were two genes (*ATF3*, *STC2*), where TGFβ1 increased expression in both, and five genes which were essentially unaffected (*FURIN*, *MAPK8*, *SMAD1*, *RHOA*, *ID3)*. Notwithstanding these gene-specific effects, the overall outcome of TGFβ1 addition was to largely eliminate the differential expression between the two cultures, as shown by the reduction in fold-differences (DSLD:NA Paso) in Table 2 relative to Table 1 (and on “**Fig 2B”** vs “F**ig 2A****”**). For the 18 genes (*PTK2B*, *MAPK14*, *ACVRL1*, *NFIB*, *EPHB2*, *HMOX1*, *SMAD6*, *ID2*, *PPARA*, *ENG*, *CREBBP*, *NFKBIA*, *BRD2*, *TGFBR2*, *MMP2*, *CDKN1B*, *EP300*, *SMAD3*) where addition of TGFβ1 was markedly inhibitory in both cultures (“**Table 2”**), most striking was the finding that the degree of inhibition was substantially greater in ADSCs from NA-Pasos than DSLD-Pasos (for example, PTK2B expression was reduced about 15-fold in NA-Pasos and 4-fold in DSLD-Pasos by TGFβ addition).

**Table 2:** Effect of TGFβ1 addition on expression of TGFβ signaling target genes in ADSCs from DSLD-Pasos and NA-Pasos.

	mRNA Abundance^1^		Fold-change with TGFβ1^2^		Fold Difference^3^
*Genes*	DSLD Paso	NA Paso	DSLD Paso	NA Paso	DSLD Paso:NA Paso
*PTK2B*	0.15	0.23	-4.13	-14.78	-1.53
*ATF3*	5.41	5.47	8.07	1.65	-1.01
*MAPK14*	1.86	3.29	-4.81	-13.06	-1.77
*ME2*	3.47	8.86	-1.57	-2.77	-2.55
*ACVRL1*	0.05	0.14	-5.80	-8.50	-2.80
*NFIB*	8.46	18.29	-2.22	-4.25	-2.16
*EPHB2*	2.83	6.39	-3.16	-5.47	-2.26
*HMOX1*	15.33	14.8	-1.95	-6.93	1.04
*SMAD6*	1.92	2.91	-2.55	-5.50	-1.52
*FOS*	8.49	3.21	1.94	-4.33	2.64
*GLI2*	0.25	0.58	-1.84	-2.53	-2.32
*STC2*	25.12	41.5	4.45	2.39	-1.65
*ID2*	19.91	23.66	-2.35	-6.02	-1.19
*PPARA*	1.07	2.22	-2.63	-3.84	-2.07
*ENG*	22.4	42.31	-1.78	-2.84	-1.89
*CREBBP*	5.47	8.82	-2.28	-4.20	-1.61
*NFKBIA*	6.41	9.02	-1.61	-3.39	-1.41
*BRD2*	24.8	33.3	-1.28	-2.82	-1.34
*TGFBR2*	12.9	24.11	-2.86	-4.46	-1.87
*MMP2*	170.2	234.2	-1.84	-3.86	-1.38
*CDKN1B*	8.37	10.3	-1.69	-3.93	-1.23
*EP300*	4.39	8.16	-2.06	-3.15	-1.86
*FURIN*	1.39	2.28	1.32	-1.28	-1.64
*MBD1*	2.36	5.49	-1.61	-1.88	-2.33
*IFRD1*	12.2	19.18	1.70	1.04	-1.57
*SMAD3*	8.64	7.47	-2.54	-6.89	1.16
*BCL2L1*	4.35	4.63	-1.33	-2.82	-1.06
*MAPK8*	4.41	7.3	1.00	-1.32	-1.66
*SMAD1*	7.13	13.29	-1.22	-1.40	-1.86
*RHOA*	142.2	204.6	1.03	-1.43	-1.44
*BACH1*	6.56	9.67	-1.50	-2.08	-1.47
*MYC*	19.23	48.95	-1.72	-1.33	-2.55
*PAI-1*	648.1	869.8	1.84	1.27	-1.34
*ID3*	239.3	292.4	1.12	-1.42	-1.22
*SNAI1*	67.9	50.54	1.34	-1.73	1.34

^1^Mean abundance data for NA-Pasos (n = 5) and DSLD-Pasos (n = 6) (with TGFβ treatment) is provided (see S1 Table for animal details).

^2^Fold-change relative to values in Table 1.

^3^ Fold difference in transcript abundance in TGFβ1-treated cultures (columns 1 and 2). Genes are listed in the same order as in Table 1. Calculation of statistical significance and abundance values are given in the Methods Section. Fold change with TGFβ, for all genes in the array is shown on heat map (**Fig 2A and 2B**).

However, the generalized inhibition of expression of TGFβ target genes by added TGFβ1 is very unexpected, suggesting that the abundance of transcript measured at 24h in these cultures might be determined not only by the cell source and the concentration of TGFβ1, but also by other considerations, such as the medium composition and the treatment period. Indeed, in a separate experiment (done with cells previously stored under liquid N2) it was found (S5 Table) that the lower transcript abundance in DSLD cells (Table 1) was reproduced in 24 h cultures (although the differentials were less clear) However, the differential was no longer detected at 48h, due to an increase in transcripts in DSLD cells relative to NA-Paso cells (data not shown). It therefore seems possible that the lower transcripts in DSLD cells at 24h (Table 1) results from an inherent delay in the 24h accumulation of transcripts of these genes under the baseline conditions of culture used here (see details of switch from medium A to medium B in Methods). It is of course also possible that transcript abundance in DSLD and/or NA-Paso cells, is very high at early stages post TGFβ1 addition (i.e. 1–4h) and that the 24h levels might be determined by the rate of removal for translation. In addition differences in TGFβ-mediated effects might result from a) modified turnover of TGFβ receptor proteins and/or associated kinase activities, b) altered levels of TGFβ “companion” proteins such as WFIKKN1 [23]) and/or c) over-production of TGFβ1 signaling antagonists such as decorin or IL-10 [24]).

### Inhibition of TGFβR1/R2 with LY2109761 in TGFβ1-treated cultures

To determine if the TGFβ1-mediated changes in mRNA levels were due to signaling via the TGFβR1/R2 receptor complex we examined the effect of addition of the dual TGFβR1/R2 kinase inhibitor LY2109761 [25, 26], together with TGFβ1, for 24h. The results (Table 3 and heat map “**Fig 2B** and **Fig 2C”**) showed that in the presence of TGFβ1+inhibitor for 24h, mRNA transcript levels were maintained at or above the “no addition” controls (shown in “**Table 1”**) for 18 and 34 of 35 genes in NA-Paso and DSLD cultures, respectively. Increases over control in mRNA (shown for all genes in DSLD cells (except *MYC* and *SNAI1*), but for only two genes (*FOS*, *TGFBR2*) in NA-Pasos) indicated that in DSLD cells (but not NA-Pasos) LY2109761 not only blocked the effect of the exogenous TGFβ1, but also overcame the apparent delay in transcript accumulation (see above). It should also be noted that the unusual stimulatory effects of TGFβ1 on transcript abundance for *STC2* and *ATF3* in cells from both horse groups, were also blocked by LY2109761, fully supporting its specificity of action at the concentration used here.

**Table 3:** Effect of LY2109761 on TGFβ1 induced changes in transcript abundance of TGFβ1 signaling target genes in DSLD-Pasos and NA-Pasos.

Genes	mRNA Abundance^1^		Fold Change ^2^		Fold Difference^3^
	(TGFβ1+LY2109761)		(TGFβ1+LY2109761:TGFβ1)		
	DSLD Paso	NA Paso	DSLD Paso	NA Paso	DSLD Paso:NA Paso
*PTK2B*	2.66	3.29	17.76	14.31	1.23
*ATF3*	0.79	3.67	-6.88	-1.49	4.66
*MAPK14*	29.85	46.40	16.05	14.10	1.55
*ME2*	18.91	21.03	5.45	2.37	1.11
*ACVRL1*	1.15	2.03	22.93	14.52	1.77
*NFIB*	36.18	75.06	4.28	4.10	2.07
*EPHB2*	31.30	29.53	11.06	4.62	-1.06
*HMOX1*	85.41	102.93	5.57	6.95	1.21
*SMAD6*	15.24	11.82	7.94	4.06	-1.29
*FOS*	10.70	23.67	1.26	7.37	2.21
*GLI2*	0.84	1.27	3.35	2.19	1.52
*STC2*	7.35	13.77	-3.42	-3.01	1.88
*ID2*	119.71	98.05	6.01	4.14	-1.22
*PPARA*	5.04	7.42	4.71	3.34	1.47
*ENG*	49.41	55.38	2.21	1.31	1.12
*CREBBP*	23.25	29.43	4.25	3.34	1.27
*NFKBIA*	23.63	24.23	3.69	2.69	1.02
*BRD2*	54.19	73.51	2.19	2.21	1.36
*TGFBR2*	98.17	160.95	7.61	6.68	1.64
*MMP2*	544.85	657.55	3.20	2.81	1.21
*CDKN1B*	27.67	33.76	3.31	3.28	1.22
*EP300*	14.32	19.97	3.26	2.45	1.39
*FURIN*	1.33	1.53	-1.05	-1.49	1.15
*MBD1*	8.91	9.47	3.78	1.72	1.06
*IFRD1*	9.83	12.16	-1.24	-1.58	1.24
*SMAD3*	35.19	45.88	4.07	6.14	1.30
*BCL2L1*	12.89	11.29	2.96	2.44	-1.14
*MAPK8*	7.96	8.60	1.81	1.18	1.08
*SMAD1*	16.97	18.67	2.38	1.40	1.10
*RHOA*	302.47	282.66	2.13	1.38	-1.07
*BACH1*	14.59	20.28	2.22	2.10	1.39
*MYC*	32.81	63.36	1.71	1.29	1.93
*PAI-1*	389.13	447.97	-1.67	-1.94	1.15
*ID3*	251.33	285.67	1.05	-1.02	1.14
*SNAI1*	37.29	40.14	-1.82	-1.26	1.08

^1^Mean abundance data for NA-Pasos (n = 5) and DSLD-Pasos (n = 6) is provided (see S1 Table for animal details).

^2^ Fold difference in transcript abundance in TGFβ1-treated cultures in the presence and absence of LY2109761.

^3^Fold difference in transcript abundance in TGFβ1 +LY2109761 treated DSLD-Paso vs. TGFβ1 +LY2109761 treated NA-Paso cultures. Genes are listed in the same order as in Table 1

### Inhibition of TGFβR1/R2 with LY2109761 in ADSCs from DSLD-Pasos and NA-Pasos without TGFb1 addition

To investigate our hypothesis that LY2109761 overcomes an apparent delay in transcript accumulation in DSLD cells (in the presence of TGFβ1) we decided to examine its effect when added in the absence of TGFβ1. ADSC cultures used for these specific experiments were established from previously stored cell preparations (see Methods for detail). We first compared the 24h transcript levels of the 35 genes in primary cultures (Table 1) with those obtained in 24h cultures of stored cells, and results (for both DSLD and NA-Paso cells) are presented as fold-difference in expression between the primary and stored cells (**S4 Table**). This showed that for most genes (in both DSLD and NA-Paso cells) the transcript levels were similar, (less than 2-fold difference for 29 of 35 DSLD genes and 26 of 35 NA-Paso genes) confirming that the expression differences between the cells in primary cultures (**Table 1**) can be largely reproduced in 24h cultures of stored cells. The effect of the TGFβR1/2 inhibitor on expression levels at 24h (with stored cells) is presented as fold-effect values (LY210976: None) in **Table 4**. This showed that relatively few genes were expressed at levels significantly (p<0.05) greater than the “None” control in either DSLD-Paso or NA-Paso cells, indicating that the enhancing effect of LY210976 on DSLD cells is operative only in the presence of exogenous TGFβ1.

**Table 4:** Fold change in transcript abundance in DSLD-Paso and NA-Paso Cells treated with LY2109761.

Gene	Fold Change in Transcript Abundance	
	(LY2109761: None)	
	DSLD Paso^1^	NA Paso^1^
*PTK2B*	2.11*	2.12*
*ATF3*	-1.27	1.16
*MAPK14*	3.12*	1.97
*ME2*	1.11	1.27
*ACVRL1*	2.42*	-11.11*
*NFIB*	-1.28	1.28
*EPHB2*	1.52	1.73
*HMOX1*	-1.27	1.85
*SMAD6*	1.39	1.12
*FOS*	1.34	3.85*
*GLI2*	-1.20	1.37
*STC2*	-1.96	1.41
*ID2*	1.01	1.31
*PPARA*	1.20	-1.03
*ENG*	-1.54	-1.20
*CREBBP*	1.48	1.26
*NFKBIA*	1.51	1.29
*BRD2*	1.24	1.15
*TGFBR2*	2.36*	2.02
*MMP2*	1.56	1.34
*CDKN1B*	1.37	1.29
*EP300*	1.24	1.16
*FURIN*	-1.96	1.59
*MBD1*	-1.06	1.28
*IFRD1*	-1.41	-1.03
*SMAD3*	1.35	1.25
*BCL2L1*	1.13	1.99
*MAPK8*	-1.47	1.19
*SMAD1*	1.25	1.15
*RHOA*	-1.01	1.06
*BACH1*	-1.27	-1.45
*MYC*	-1.12	1.38
*PAI-1*	-2.22*	-1.52
*ID3*	1.09	1.16
*SNAI1*	-1.47	-1.25

^1^Mean abundance data for NA-Pasos (n = 2) and DSLD-Pasos (n = 2) was used (see S1 Table for animal details) to calculate fold change as described in Methods. Genes are listed in the same order as in Table 1.

(*) Significantly affected by LY2109761, p<0.05

### The effect of TGFβ1 on the expression of chromatin modification enzymes in ADSCs from NA-Pasos and DSLD-Pasos

In previous murine Achilles tendon studies [16] we demonstrated that TGFβ1 injection resulted in acute changes in the expression of many chromatin modifiers, suggesting analysis of mRNA samples from the present study might be useful. Analysis of RNA from ligament tissues (assayed for matrix genes, “**Fig 1”**) gave no evidence of a difference between DSLD-Pasos and NA-Pasos (“**S5 Table”**). Also, analysis of 24h untreated ADSCs (from “**Table 1”**) showed that the basal expression of the modification enzymes was very similar for DSLD-Pasos and NA-Pasos (“**Table 5”**), supporting the uniqueness of the specific differences found in TGFβ signaling genes (“**Table 1”**). Moreover, the expression of modifier genes was generally decreased (or unaltered) by TGFβ1 to the same extent in the two cell sources (“**Table 5”**) and this is also in contrast to the TGFβ target genes which were generally more inhibited by TGFβ1 in NA-Pasos than in DSLD-Pasos **(“Table 2”).**

**Table 5:** Effect of TGFβ1 on expression of chromatin-modifying enzymes in ADSCs.

Gene Groups	Genes	Fold Difference	Fold TGFβ1: NONE	
		DSLD Paso:NA Paso	DSLD Paso	NA Paso
**DHD**	*KDM1A*	-1.12	-2.02	-1.37
**DM**	*DNMT1*	-1.52	-1.30	-1.16
	*DNMT3B*	-1.06	-5.11	-3.48
**HA**	*ATF2*	-1.23	-1.53	-1.83
	*CSRP2BP*	-1.13	-2.06	-1.30
	*ESCO1*	1.04	-3.00	-2.68
	*HAT1*	1.18	1.25	-1.01
	*KAT2A*	-1.16	-1.87	-1.55
	*KAT2B*	1.11	-5.31	-5.28
	*KAT7*	-1.09	-2.32	-1.27
	*HDAC1*	-1.16	-1.82	-1.66
**HD**	*HDAC11*	1.65	-20.40	-4.43
	*HDAC2*	1.08	-1.52	-1.19
	*HDAC3*	-1.01	-3.01	-2.06
	*HDAC6*	1.07	-5.38	-2.38
	*AURKA*	-2.01	1.06	1.12
**HM**	*AURKB*	-2.04	1.22	1.06
	*CARM1*	-1.20	-1.78	-1.65
	*PRMT1*	-1.48	1.17	1.09
	*PRMT3*	1.07	-1.11	-1.16
	*PRMT5*	-1.18	1.08	1.06
	*PRMT6*	-1.16	-1.42	-1.32
	*PRMT7*	1.02	-2.12	-1.39
	*SUV39H1*	-1.56	1.88	1.73
	*DZIP3*	1.18	-4.27	-2.49
**HP**	*NEK6*	1.12	-1.37	1.17
	*PAK1*	1.06	-1.41	-1.72
	*RNF20*	1.15	-50.23	-33.03
	*SETD1A*	-1.40	-5.01	-3.07
**HU**	*SETD8*	1.04	-8.11	-4.17
	*USP16*	1.07	57.89	114.02
	*USP22*	1.04	-25.50	-11.66
	*WHSC1*	-1.38	-1.00	-1.03

DHD, DNA/Histone Demethylases; DM, DNA methylases; HA, histone acetylases; HD, Histone Deacetylases; HM, Histone Methylases; HP, Histone Phosphorylases; HU, Histone Ubiquitinases. Mean abundance data for NA-Pasos (n = 5) and DSLD-Pasos (n = 6) is provided (see S1 Table for animal details). SD values are in parentheses

## Discussion

While research into the molecular pathology of DSLD has focused on structural extracellular matrix components, such as decorin [27] and aggrecan [1, 5], the studies described here indicate that the pathology is accompanied by a more general metabolic disturbance, characterized by an altered expression/activity of TGFβ-signaling target genes in fibroblastic type cells. While precise details of the metabolic disturbance are unknown, the most definitive data linking the DSLD pathology to TGFβ1-signaling is the approximate 4-fold deficiency (fold-difference in DSLD-Paso /NA-Paso about minus 4) in transcript abundance for 19 signaling target genes (*PTK2B*, *ATF3*, *MAPK14*, *ME2*, *ACVRL1*, *NFIB*, *EPHB2*, *HMOX1*, *SMAD6*, *FOS*, *GLI2*, *STC2*, *ID2*, *PPARA*, *ENG*, *CREBBP*, *NFKBIA*, *BRD2*, *TGFBR2*) in adipose-derived stromal fibroblasts (see Table 1). It should be noted that the 4-fold difference was based on mean abundance values from six DSLD-Pasos and five NA-Pasos, so that a comparison based on single horses may result in more or less pronounced differences. It should also be noted that this difference was observed under very specific culture conditions involving a switch from DMEM/10%FBS to AMEM/5%FBS for the final 24h and could be explained, for example, by a delay in transcript accumulation in DSLD cells following the medium change (as discussed in Results).

Independent of these considerations, the significance of a widely reduced transcript abundance of TGFβ target genes in DSLD-Paso cells at 24h is underlined by the fact that the expression of at least 31 chromatin modifying enzymes was essentially identical in DSLD and NA-Paso cells under the same conditions (S6 Table). Such a specific alteration in the TGFβ signaling pathway is entirely in keeping with the pathology of the DSLD-affected ligament, namely extensive scar-like remodeling of the ligament itself (“**S1 Fig”),** and altered matrix proteoglycan composition [5, 27]. Indeed the proteoglycan changes observed, including increased total aggrecan [5] and 6-sulfation of N-acetyl galactosamine [27] are established downstream effects of altered TGFβ signaling in connective tissues in general [7, 28–31]. However, the detailed molecular mechanism underlying the altered TGFβ1 responses in the affected horses remain to be established. Additional genetic components including epigenomic regulations of gene expression may provide a predisposition to disease development. Since the Pasos are training for competition from about 2–3yrs onward, it seems possible that horses will exhibit a variable response to different training protocols, procedures of care, breeding, diet and aging methods so that some succumb to DSLD whereas others do not.

In addition to suggesting new approaches (such as ADSC cultures) in tendinopathy research the results presented here might form the basis of a gene expression test for DSLD susceptibility in Peruvian Paso horses of breeding-age and either sex. Such a test would require the isolation and culture of ADSCs under defined subculture and medium conditions, followed by quantitative analysis of TGFβ target gene expression on commercially available array plates. At present, a definitive result would require replicate analysis of cells from the patient horse and (in parallel) cells from a non-affected Paso horse (preferably at least 15yrs old). In essence, a diagnosis of DSLD-positive would follow if the transcript abundance of the 35 genes listed in Table 1 is more than 1.5-fold lower in the patient cells than the control cells. The extent to which this test could be used prognostically in young horses is unknown at present, however this will require prospective testing of ADSCs from growing Peruvian Pasos, followed by continuous clinical evaluation.

The in vitro study described here does not definitively implicate component(s) of the TGFβ signaling pathway in the heritable ‘genetic’ elements underlying the development of DLSD disease in Paso horses. Without data on specific genomic or epigenomic modifications in the Paso strain, the TGFβ pathway cannot be considered a therapeutic target. However, application of the reported methodology may prove useful in determining those Paso horses with a susceptibility to DLSD before the onset of clinical symptoms.

While transcript profiling of ADSCs for diagnostic purposes has not been in wide use it has been shown to distinguish individuals with Type II diabetes from healthy controls [32], and also to help explain why ADSCs from diabetic patients have a reduced wound healing capacity on implantation. In addition, Sporadic Multiple Symmetric Lipomatosis has been diagnosed by transcript analysis of patient-derived ADSCs for genes associated with proliferation, hormonal regulation, and mitochondria [33]. Since allogeneic blood-derived MSCs have been used in an attempt to treat DSLD in a Dutch Warmblood [34], application of the TGFβ expression assay reported here (with ADSCs) could be extended to non-invasively obtained blood-borne fibrocytes [35, 36] for both diagnostic and cell-therapy purposes.

The in vitro study described here does not definitively implicate component(s) of the TGFβ signaling pathway in the heritable ‘genetic’ elements underlying the development of DLSD disease in Paso horses. Without additional data on specific genomic or epigenomic modifications in the Paso strain, the TGFβ pathway cannot be considered a therapeutic target. However, application of the reported methodology may prove useful in determining those Paso horses with a susceptibility to DLSD before the onset of clinical symptoms.

In conclusion, the following limitations of the vitro study described above should be noted: Firstly a definitive implication of one or more genes in the TGFβ signaling pathway in the heritable components underlying the development of DLSD disease in Paso horses cannot be concluded. Secondly, without additional investigations of specific genomic or epigenomic modifications in the Paso strain, the TGFβ pathway cannot be considered a therapeutic target. Although we did not detect any significant differences in expression of DNA/histone modification genes between normal and diseased tissues or in ADSC cell preparations from affected and non-affected Paso horses, a modified ligament epigenome during the period of disease development cannot be excluded. Indeed this should be considered an important question for future studies.

## Supporting Information

S1 Fig (A) Suspensory ligament tissues from Normal, DSLD-Paso and NA Paso used for transcriptomic analyses. (B) Typical Ultrasound Images from a Normal and a DSLD Paso; region of pathologically remodeled collagen fibrils is marked by an arrow.
(TIF)

S2 Fig **Morphologies of primary ADSC cultures form DSLD-Paso (a) and NA-Paso (b) horses.** Untreated cells (NONE, top LH panels) show typical appearance of fibroblastic phenotype. Cells respond to a 24h exposure to 10 ng/mL TGFβ1 (in AMEM/5% FCS) by contraction of cell layers (bottom LH panels). Addition of LY2109761 inhibits TGFβ1-induced contraction (bottom RH panels), but does not affect morphological appearance of untreated cultures (top RH panels).
(TIF)

S1 Table Details of Horses used in this study. (PDF)

S2 Table Custom QPCR Array for TGFβ1 Signaling Target Genes. (PDF)

S3 Table Custom QPCR Array for Chromabtin Modification Genes. (PDF)

S4 Table Effect of Cell Storage on Transcript Abundance of TGFβ Signaling Target Genes in ADSC cultures. ^**1**^Cultures were established from previously frozen cells and QPCR assays done on confluent layers maintained for 24h in AMEM/5% FCS medium (see Methods for details). Average Transcript Abundance was calculated from triplicate cultures from 2 DSLD-Paso and 2 NA-Paso horses (see Table 1 for animal details). ^**2**^Genes are listed in the same order as in Table 1.
(PDF)

S5 Table Expression of chromatin-modifying enzymes in ligaments. ^**1**^Gene groupings as defined in S3 Table. ^**2**^Mean abundance data (standard deviation) for NA-Pasos (n = 9) and DSLD-Pasos (n = 9) is provided (see S1 Table for animal details).
(PDF)

S6 Table Effect of TGFβ1 on expression of chromatin-modifying enzymes in ADSCs. ^**1**^Gene groups as described in S3 Table. ^**2**^Values represent the mean of duplicate cultures from DSLD-Pasos (n = 6) and NA Paso Horses n = 5 (see Table 1 for animal details).
(PDF)

## Abbreviations

DSLD: degenerative suspensory ligament disease
DSLD-Paso: DSLD-affected Peruvian Paso horse
SL: Suspensory ligament
NA-Paso: Nonaffected Peruvian Paso horse
Non-Pasos: Non-affected non-Paso horses
ADSCs: Adipose-derived stromal fibroblasts
TGFβ: Transforming Growth Factor beta
