# Supplementary figures and images for: Degenerative Suspensory Ligament Desmitis (DSLD) in Peruvian Paso Horses Is Characterized by Altered Expression of TGFβ Signaling Components in Adipose-Derived Stromal Fibroblasts
Source: PLoS One. 2016 Nov 30;11(11):e0167069. doi: 10.1371/journal.pone.0167069 (PMC5130251; doi:10.1371/journal.pone.0167069)

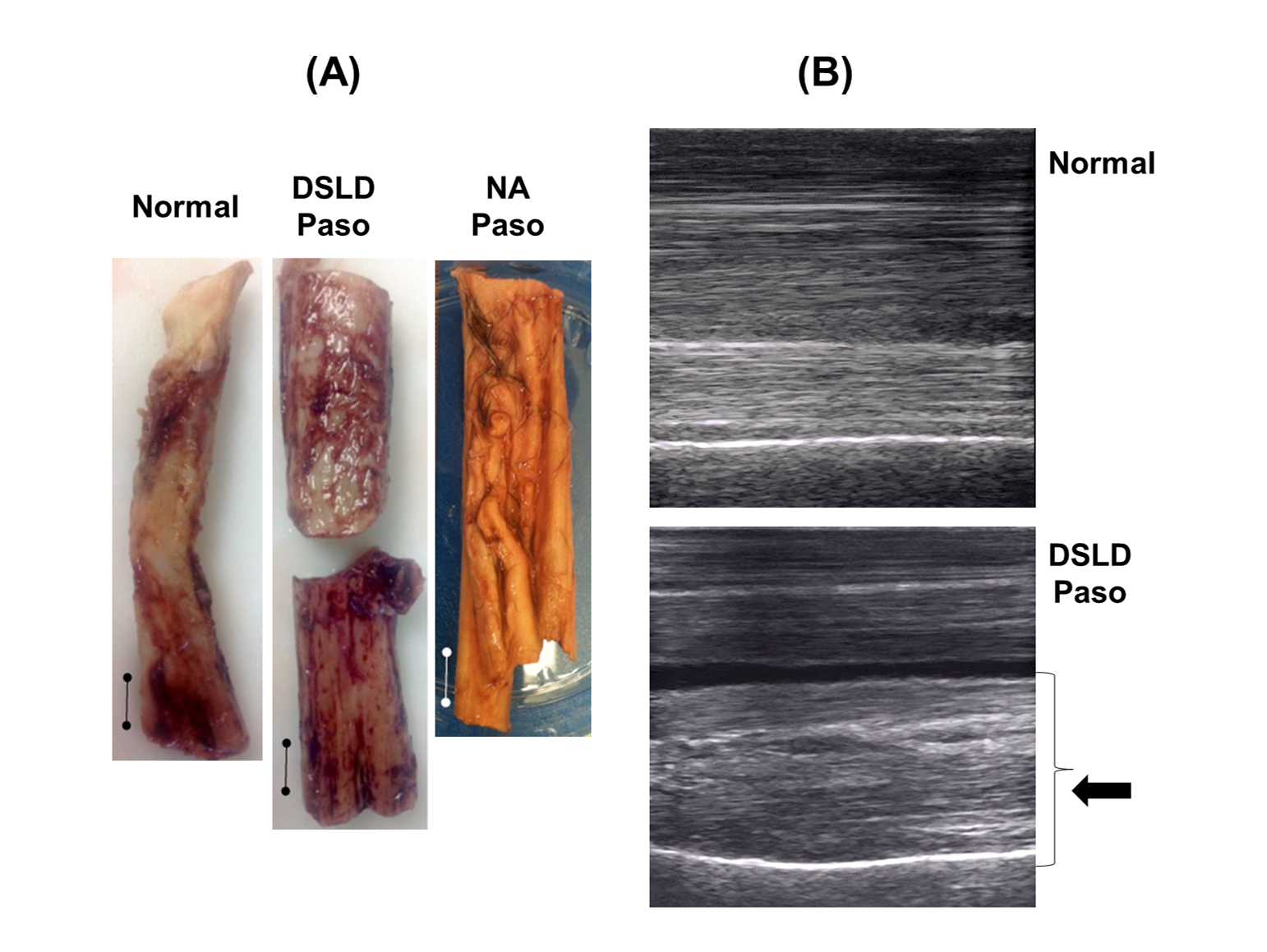

Supplement: S1 Fig — (A) Suspensory ligament tissues from Normal, DSLD-Paso and NA Paso used for transcriptomic analyses. (B) Typical Ultrasound Images from a Normal and a DSLD Paso; region of pathologically remodeled collagen fibrils is marked by an arrow. (TIF) [file pone.0167069.s001.tif]

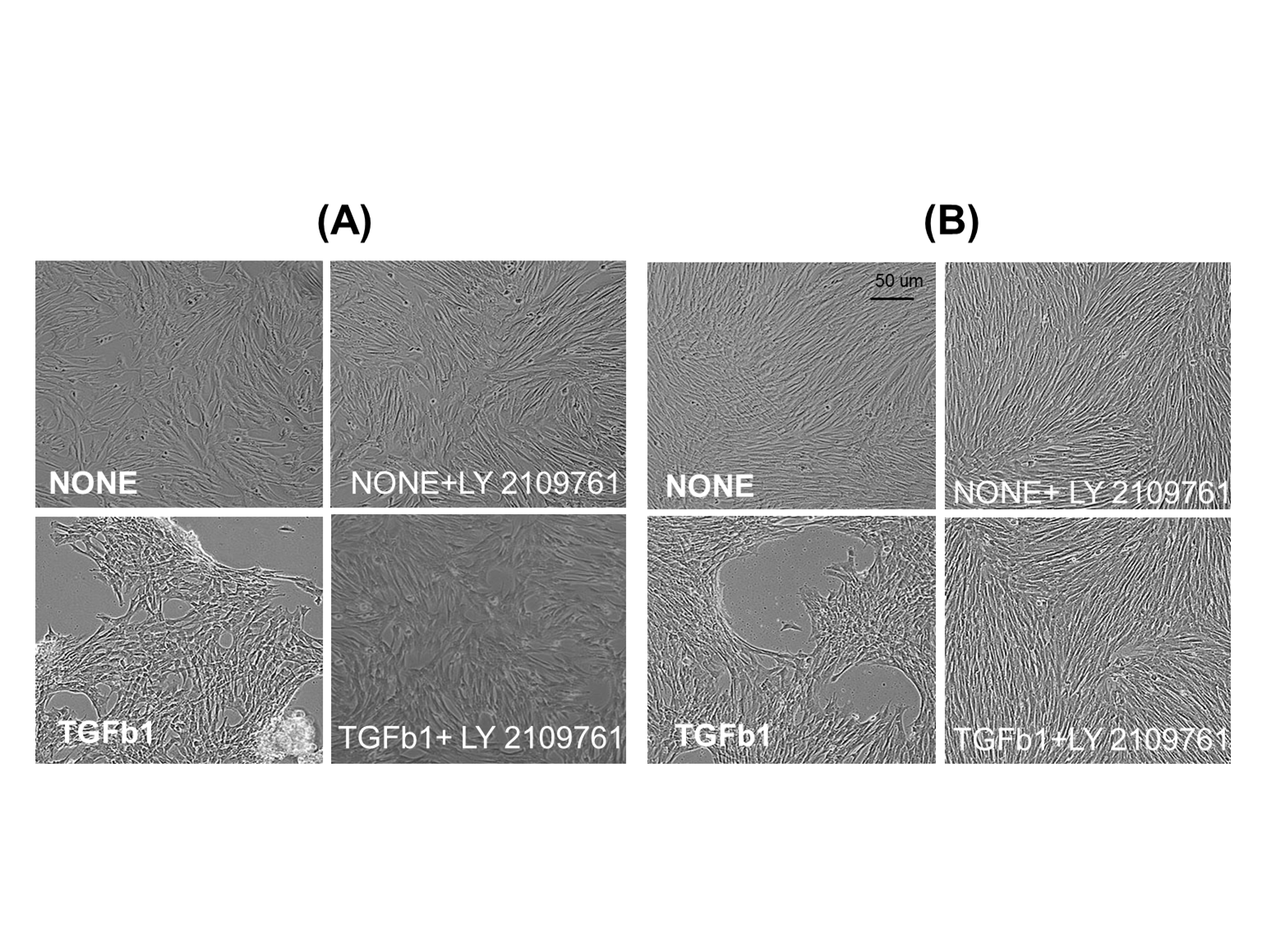

Supplement: S2 Fig — Morphologies of primary ADSC cultures form DSLD-Paso (a) and NA-Paso (b) horses. Untreated cells (NONE, top LH panels) show typical appearance of fibroblastic phenotype. Cells respond to a 24h exposure to 10 ng/mL TGFβ1 (in AMEM/5% FCS) by contraction of cell layers (bottom LH panels). Addition of LY2109761 inhibits TGFβ1-induced contraction (bottom RH panels), but does not affect morphological appearance of untreated cultures (top RH panels). (TIF) [file pone.0167069.s002.tif]
