# Supplementary material for: Degenerative Suspensory Ligament Desmitis (DSLD) in Peruvian Paso Horses Is Characterized by Altered Expression of TGFβ Signaling Components in Adipose-Derived Stromal Fibroblasts
Source: PLoS One. 2016 Nov 30;11(11):e0167069. doi: 10.1371/journal.pone.0167069 (PMC5130251; doi:10.1371/journal.pone.0167069)
Supplement: S1 Table — (PDF) [file pone.0167069.s003.pdf]

<u>Table S1:</u> Details of Horses used in this study

Non Affected Peruvian Pasos	Horse #	Breed	Sex	Age (years)	Assay
	1	Peruvian Paso	Gelding	19	ADSC
	2	Peruvian Paso	Stallion	18	ADSC
	3	Peruvian Paso	Gelding	18	ADSC; Frozen-ADSC;
	4	Peruvian Paso	Mare	26	ADSC; SLT
	5	Peruvian Paso	Mare	14	Frozen-ADSC; ADSC; SLT
**DSLD Affected Peruvian Pasos**					
	6	Peruvian Paso	Gelding	18	ADSC; Frozen-ADSC;SLT
	7	Peruvian Paso	Gelding	9	ADSC
	8	Peruvian Paso	Gelding	5	ADSC
	9	Peruvian Paso	Mare	14	ADSC; Frozen-ADSC;
	10	Peruvian Paso	Mare	12	ADSC
	11	Peruvian Paso	Mare	8	ADSC
	12	Peruvian Paso	Mare	15	SLT
	13	Peruvian Paso	Mare	13	SLT
	14	Peruvian Paso	Mare	21	SLT
**Normal horses**					
	15	Arabian	Gelding	31	SLT
	16	Arabian	Gelding	19	SLT
	17	Appaloosa	Gelding	11	SLT
	18	Thoroughbred	Mare	26	SLT
	19	Thoroughbred	Mare	25	SLT
	20	Irish warmblood	Mare	8	SLT
