# Supplementary material for: Degenerative Suspensory Ligament Desmitis (DSLD) in Peruvian Paso Horses Is Characterized by Altered Expression of TGFβ Signaling Components in Adipose-Derived Stromal Fibroblasts
Source: PLoS One. 2016 Nov 30;11(11):e0167069. doi: 10.1371/journal.pone.0167069 (PMC5130251; doi:10.1371/journal.pone.0167069)
Supplement: S2 Table — (PDF) [file pone.0167069.s004.pdf]

<u>Table S2:</u> Custom QPCR array for equine TGFb1-signaling target genes

Signal Transduction:

ACLY, ACVRL1, ATF4, BACH1, BHLHE40, BRD2, CREBBP, E2F4, ENG, EP300, FOS, GLI2, GTF2I, HEY1,

HSP90AA1, ID1, ID2, ID3, MBD1, ME2, NFIB, NOTCH1, RBL1, RUNX1, RYBP, SMAD1, SMAD3, SMAD5,

SMAD6, SNAI1, SP1, SREBF2, STC2, TGFB2, TGFBR2,

Cell Migration and Differentiation:

ACTA2, ACVR1, AR, ATF3, CDC6, CDKN1B, EMP1, EPHB2, FN1, FURIN, HES1, HMOX1, IFRD1, MAPK14, MMP2, MYC, PAI-1, PDGFA, PPARA, PTGS2, PTHLH, PTK2, PTK2B, THBS1, VEGFA

Apoptosis

BCL2L1, BDNF, CEBPB, CREB1, CRYAB, CTNNB1, GADD45B, HERPUD1, HMOX1, KLF10, MAP3K7, MAPK8, MSX2, NFKBIA, RAD21, RHOA, RHOB, TNFSF10
