# Supplementary material for: Degenerative Suspensory Ligament Desmitis (DSLD) in Peruvian Paso Horses Is Characterized by Altered Expression of TGFβ Signaling Components in Adipose-Derived Stromal Fibroblasts
Source: PLoS One. 2016 Nov 30;11(11):e0167069. doi: 10.1371/journal.pone.0167069 (PMC5130251; doi:10.1371/journal.pone.0167069)
Supplement: S3 Table — (PDF) [file pone.0167069.s005.pdf]

Table S3: Custom QPCR array for equine chromatin modification genes

DNA/Histone Demethylase:

KDM1A

DNA Methylases:

DNMT1, DNMT3B

Histone Acetylases

ATF2, CIITA, CSRP2BP, ESCO2, HAT1, KAT2A, KAT2B, KAT7

Histone Demethylases:

HDAC1, HDAC11, HDAC2, HDAC3, HDAC6

Histone Methylases:

CARM1, PRMT1, PRMT3, PRMT5, PRMT6, PRMT7, SUV39H1, AURKA, AURKB

Histone Phosphorylases:

NEK6, PAK1, DZIP3, RNF20

Histone Ubiquitinases:

USP16, USP22, SETD1A, SETD8, WHSC1
