# Supplementary material for: Degenerative Suspensory Ligament Desmitis (DSLD) in Peruvian Paso Horses Is Characterized by Altered Expression of TGFβ Signaling Components in Adipose-Derived Stromal Fibroblasts
Source: PLoS One. 2016 Nov 30;11(11):e0167069. doi: 10.1371/journal.pone.0167069 (PMC5130251; doi:10.1371/journal.pone.0167069)
Supplement: S4 Table — 1Cultures were established from previously frozen cells and QPCR assays done on confluent layers maintained for 24h in AMEM/5% FCS medium (see Methods for details). Average Transcript Abundance was calculated from triplicate cultures from 2 DSLD-Paso and 2 NA-Paso horses (see Table 1 for animal details). 2Genes are listed in the same order as in Table 1. (PDF) [file pone.0167069.s006.pdf]

<u>Table S4:</u> Effect of cell storage on transcript abundance of TGFb-signaling target genes in ADSC cultures

**Genes**	**Fold difference (Stored cells :Primary cells)**	
	DSLD Paso	NA Paso
	**24h[1]**	**24h[1]**
PTK2B[4]	1.42	-1.68
ATF3	2.36	-2.11
MAPK14	-1.49	-2.17
ME2	2.39	-1.39
ACVRL1	2.07	1.36
NFIB	1.48	-1.89
EPHB2	1.37	-2.13
HMOX1	-1.42	-3.92
SMAD6	1.48	1.06
FOS	-1.43	-3.66
GLI2	1.17	-1.12
STC2	2.02	-3.62
ID2	-1.06	-1.56
PPARA	1.67	1.18
ENG	1.34	-1.76
CREBBP	1.21	-1.40
NFKBIA	-1.04	-2.05
BRD2	1.60	-1.36
TGFBR22	-1.15	-1.31
MMP2	-1.24	-2.68
CDKN1B	1.45	-1.51
EP300	1.23	-1.52
FURIN	-1.36	-2.41
MBD1	2.16	1.11
IFRD1	1.22	-1.73
SMAD3	1.02	1.06
BCL2L1	1.40	-1.54
MAPK8	1.07	-1.36
SMAD1	1.17	-1.15
RHOA	1.88	-1.01
BACH1	1.10	-1.56
MYC	1.12	-1.43
PAI-1	2.58	1.24
ID3	1.23	1.01
SNAI1	-1.23	-1.34

[1] Fold difference in transcript abundance in stored cells vs primary cultures. (see Methods section on isolation and storage of adipose-derived stromal fibroblasts (ADSCs) for details). Mean transcript abundance in stored cells was calculated from triplicate cultures of 2 DSLD-Paso and 2 NA-Paso horses (see Table S1 for animal details). [4]Genes are listed in the same order as in Table 1.
