# Supplementary material for: Degenerative Suspensory Ligament Desmitis (DSLD) in Peruvian Paso Horses Is Characterized by Altered Expression of TGFβ Signaling Components in Adipose-Derived Stromal Fibroblasts
Source: PLoS One. 2016 Nov 30;11(11):e0167069. doi: 10.1371/journal.pone.0167069 (PMC5130251; doi:10.1371/journal.pone.0167069)
Supplement: S5 Table — 1Gene groupings as defined in S3 Table. 2Mean abundance data (standard deviation) for NA-Pasos (n = 9) and DSLD-Pasos (n = 9) is provided (see S1 Table for animal details). (PDF) [file pone.0167069.s007.pdf]

Table S5 Expression of chromatin-modifying enzymes in ligaments

**Gene Group**[1]		**Abundance**[2]			
		DSLD Paso		NAPaso	
**DHD**	*KDM1A*	42.63	(20.43)[2]	35.25	(4.93)[2]
**DM**	*DNMT1*	5.46	(3.46)	5.87	(1.92)
	DNMT3B	1.44	(0.84)	0.9	(0.15)
**HA**	*ATF2*	43.28	(15.69)	28.8	(9.53)
	CSRP2BP	3.98	(2.02)	4.84	(2.49)
	ESCO2	10.78	(3.94)	7.99	(2.89)
	HAT1	26.46	(12.42)	28.31	(4.43)
	KAT2A	14.04	(7.59)	10.08	(2.76)
	KAT2B	95.02	(29.70)	70.75	(11.19)
	KAT7	28.18	(9.18)	21.7	(6.67)
	HDAC1	45.90	(15.28)	47.47	(34.21)
**HD**	*HDAC11*	5.83	(3.18)	5.71	(0.58)
	HDAC2	50.67	(27.94)	37.9	(13.76)
	HDAC3	15.31	(4.59)	12.89	(3.72)
	HDAC6	8.54	(2.30)	8.99	(2.77)
	AURKA	4.86	(1.52)	3.74	(0.23)
**HM**	*AURKB*	0.19	(0.18)	0.12	(0.08)
	CARM1	18.81	(6.55)	19.6	(3.78)
	PRMT1	46.38	(22.24)	53.54	(5.76)
	PRMT3	13.93	(5.02)	15.46	(2.33)
	PRMT5	31.70	(9.81)	29.81	(2.35)
	PRMT6	6.93	(2.35)	5.84	(1.65)
	PRMT7	8.19	(4.45)	5.59	(1.27)
	SUV39H1	3.31	(1.39)	4.7	(1.58)
	DZIP3	26.69	(11.92)	16.53	(2.96)
**HP**	*NEK6*	14.62	(5.10)	17.37	(8.04)
	PAK1	13.55	(6.34)	12.57	(2.61)
	RNF20	30.48	(10.37)	20.63	(4.57)
	SETD1A	9.52	(4.02)	8.91	(3.75)
**HU**	*SETD8*	19.52	(6.82)	13.49	(0.79)
	USP16	1.57	(0.53)	0.68	(0.29)
	USP22	86.15	(40.2)	80.77	(6.11)
	WHSC1	4.91	(1.91)	3.63	(0.98)

[1] Gene groupings as defined in Table S3. **[2]**Mean abundance data (standard deviation) for NA-Pasos (n=9) and DSLD-Pasos (n=9) is provided (see Table S1 for animal details).
