# Supplementary material for: Degenerative Suspensory Ligament Desmitis (DSLD) in Peruvian Paso Horses Is Characterized by Altered Expression of TGFβ Signaling Components in Adipose-Derived Stromal Fibroblasts
Source: PLoS One. 2016 Nov 30;11(11):e0167069. doi: 10.1371/journal.pone.0167069 (PMC5130251; doi:10.1371/journal.pone.0167069)
Supplement: S6 Table — 1Gene groups as described in S3 Table. 2Values represent the mean of duplicate cultures from DSLD-Pasos (n = 6) and NA Paso Horses n = 5 (see Table 1 for animal details). (PDF) [file pone.0167069.s008.pdf]

Table S6: Effect of TGFb1 on expression of chromatin-modifying enzymes in ADSCs

Gene Group[1]		NONE				+TGFb1			
		DSLD-Paso[2]		NA-Paso[2]		DSLD-Paso[2]		NA-Paso[2]	
**DHD**	*KDM1A*	30.23	(7.60)[3]	33.85	(8.13)	14.96	(0.87)	24.64	(14.71)
**DM**	*DNMT1*	5.28	(1.65)	8.05	(2.64)	4.05	(2.96)	6.93	(5.99)
	DNMT3B	1.38	(0.78)	1.46	(0.61)	0.27	(0.03)	0.42	(0.39)
**HA**	*ATF2*	18.66	(3.90)	22.95	(4.95)	12.16	(5.98)	12.57	(6.75)
	CSRP2BP	2.70	(0.67)	3.05	(0.75)	1.31	(0.19)	2.35	(1.19)
	ESCO1	1.68	(0.78)	1.61	(0.70)	0.56	(0.13)	0.60	(0.42)
	HAT1	28.86	(6.46)	24.37	(3.87)	36.02	(3.96)	24.18	(12.26)
	KAT2A	4.98	(1.50)	5.78	(1.75)	2.67	(0.45)	3.74	(2.24)
	KAT2B	28.03	(5.15)	25.33	(6.67)	5.28	(2.33)	4.80	(2.82)
	KAT7	13.23	(4.24)	14.47	(3.05)	5.71	(0.79)	11.40	(6.58)
	HDAC1	33.98	(7.47)	39.27	(10.60)	18.64	(0.87)	23.72	(12.61)
**HD**	*HDAC11*	1.02	(0.56)	0.62	(0.32)	0.05	(0.02)	0.14	(0.12)
	HDAC2	30.94	(9.16)	28.77	97.40)	20.40	(4.43)	24.17	(11.91)
	HDAC3	19.05	(6.38)	19.24	(5.05)	6.32	(1.16)	9.35	(5.33)
	HDAC6	21.21	(8.39)	19.77	(3.09)	3.94	(0.62)	8.30	(3.43)
	AURKA	6.85	(1.20)	13.80	(3.04)	7.23	(3.79)	15.44	(15.43)
**HM**	*AURKB*	3.95	(0.95)	8.04	(2.78)	4.80	(6.30)	8.56	(6.69)
	CARM1	26.01	(6.23)	31.30	(8.32)	14.62	(4.52)	18.93	(11.16)
	PRMT1	49.24	(12.12)	73.03	(12.69)	57.58	(13.09)	79.96	(50.66)
	PRMT3	14.33	(2.76)	13.36	(2.60)	12.91	(1.81)	11.48	(5.66)
	PRMT5	13.54	(5.32)	15.96	(1.07)	14.68	(12.18)	16.89	(7.15)
	PRMT6	3.92	(1.25)	4.54	(1.16)	2.77	(1.25)	3.44	(1.78)
	PRMT7	9.76	(2.11)	9.54	(2.13)	4.61	(0.60)	6.88	(3.25)
	SUV39H1	3.46	(1.20)	5.40	(0.90)	6.49	(2.93)	9.35	(4.51)
	DZIP3	15.67	(6.04)	13.25	(3.27)	3.67	(2.19)	5.33	(2.35)
**HP**	*NEK6*	23.74	(5.68)	21.20	(4.49)	17.34	(2.66)	24.76	(16.02)
	PAK1	56.19	(10.09)	53.22	(19.76)	39.73	(15.02)	30.98	(14.81)
	RNF20	11.05	(3.48)	9.58	(3.06)	0.22	(0.07)	0.29	(0.16)
	SETD1A	7.71	(2.79)	10.83	(3.04)	1.54	(0.83)	3.53	(2.75)
**HU**	*SETD8*	19.47	(4.34)	18.80	(2.82)	2.40	(0.50)	4.51	(2.40)
	USP16	0.44	(0.20)	0.41	90.10)	25.47	(4.19)	46.75	(28.13)
	USP22	85.68	(27.15)	82.78	(18.55)	3.36	(1.17)	7.10	(5.02)
	WHSC1	5.30	(1.67)	7.29	(1.09)	5.30	(1.67)	7.10	(5.02)

[1] Gene groups as described in Table S-3. [2]Values represent the mean of duplicate cultures from DSLD-Pasos (n= 6) and NA Paso Horses n= 6 (see Table 1 for animal details). [3]SD values are in parentheses
